# Psychometric Properties of the Arabic Version of the Drug Use Disorders Identification Test (DUDIT) in Clinical, Prison Inmate, and Student Samples

**DOI:** 10.1007/s12529-016-9623-2

**Published:** 2017-01-25

**Authors:** Anis Sfendla, Btissame Zouini, Dina Lemrani, Anne H. Berman, Meftaha Senhaji, Nóra Kerekes

**Affiliations:** 1 Department of Biology, Faculty of Sciences, Abdelmalek Essaadi University, Tetouan, Morocco; 2 Department of Clinical Neuroscience, Center for Psychiatry Research, Karolinska Institutet, Stockholm, Sweden; 3 Department of Health Sciences, University West, Trollhättan, Sweden

**Keywords:** DUDIT, Drug use, Psychometric properties, Arabic, Screening, MeSHe study

## Abstract

**Purpose:**

The study aimed to validate the Arabic version of the Drug Use Disorders Identification Test (DUDIT) by (1) assessing its factor structure, (2) determining structural validity, (3) evaluating item-total and inter-item correlation, and (4) assessing its predictive validity.

**Method:**

The study population included 169 prison inmates, 51 patients with clinical diagnosis of substance used disorder, and 53 students (*N* = 273). All participants completed the self-report version of the Arabic DUDIT. After exploratory factor analysis, internal consistency of the Arabic DUDIT was determined and external validation was performed.

**Results:**

Principal factor analysis showed that Arabic DUDIT exhibited only one factor, which explained 66.9% of the variance. Reliability based on Cronbach’s alpha was .95. When compared to the DSM-IV substance use disorder diagnosis in a clinical sample, DUDIT had an area under the curve (AUC) of .98, with a sensitivity of .98 and a specificity of .90.

**Conclusion:**

The Arabic version of DUDIT is a valid and reliable tool for screening for drug use in Arabic-speaking countries.

## Introduction

Research in the field of psychiatry, including substance use disorders, is quite rare in the Arabic world, and Morocco is no exception to this matter [1]. As Gaferi et al. [2] pointed out, there is an increased need for published research within the field of substance use from Arabic-speaking countries, where despite cultural, social, and/or religious facets, global reports indicate an increasing prevalence of mental illness in this field [3] possibly as a consequence of rapid development and modernization [2, 4, 5]. According to the latest report by the International Narcotics Control Board (2014) [6], Morocco (beside Afghanistan) is still the largest producer of cannabis resin in the world, supplying the illicit markets of western and central Europe and North Africa. This fact raises the obvious point that Morocco might also be one of the leading countries in terms of drug use or at least cannabis use. However, the prevalence of substance abuse among citizens aged 15 years and above, in the years 2004 and 2005, was 5.8% according the nationwide survey on mental health and drug addiction carried out by Morocco’s Ministry of Health [7]. This rate is 1.6 times lower than that measured in the USA [8]. A recent study concluded that young adults’ involvement in substance use in Morocco was substantially lower than the corresponding rates in Europe or the USA [9]. Importantly, these studies used the European ESPAD survey [10] and the Mini International Neuropsychiatric Interview (MINI) [11], respectively, to collect data about the prevalence of drug use/abuse. In order to be able to compare data about the true prevalence of drug use in Arabic-speaking countries to other international information, we need validated and reliable instruments with good psychometric properties. Only then will we be able to discern the underlying reasons of discrepancies in this global matrix.

The early identification of individuals with drug problems and evaluation of treatment strategies requires valid and reliable screening instruments. Several of these instruments have focused on substance use and related constructs [12, 13]. Some of the most frequently used instruments for these purposes are the Drug Abuse Screening Test (DAST) [14], the CAGE-AID (Cut-down, Annoyed, Guilty, Eye-opener–Adapted to Include Drugs) [15], the Alcohol, Smoking and Substance Involvement Screening Test (ASSIST) [16], and the Drug Use Disorders Identification Test (DUDIT) [17].

The DUDIT is one of the newest members in the above list of screening instruments. It was developed with the specific aims of assessing usage patterns and related problems, as well as identifying the risk of harmful use or dependence according to DSM-IV and ICD-10 by collecting information about drug intake and associated problems. The DUDIT has been used in European countries, such as Sweden, from where it originates [18, 19]; Norway [20, 21]; Hungary [22]; and the Netherlands [23, 24] but also used outside Europe, in the USA [25, 26], South Africa [27], and Turkey [28, 29]. In the original publication on the DUDIT, Berman and colleagues [17] showed that the instrument has good psychometric properties, such as high internal consistency both in clinical (Cronbach’s alpha .80) and in general populations (Cronbach’s alpha .93). Several studies have confirmed the strong validity of the DUDIT for assessing drug-related risk behavior and/or addiction in various samples. These samples include the general population [22, 26, 28], prisoners [17], probationers [23], offenders with mental health problems [18], patients with substance use disorder (SUD) [30] or with a diagnosis of psychosis [21], and in samples of adolescents and school-attending youths [24, 27]. With this background, the present study aims to validate and establish the psychometric properties of the Arabic version of DUDIT with the help of a clinical sample (where DSM-IV diagnoses are available), a prison sample (where substance use often companies criminal behavior), and a student sample (where the risk of substance use disorder is minimal).

## Subjects and Methods

During July 2013 and July 2014, we collected information about somatic and mental health in defined samples of Moroccans using the “Mental and Somatic Health without borders” (MeSHe) survey. The MeSHe survey, constructed by the project leader and co-author (NK), focuses on somatic and mental health profiles coupled to substance use and aggressive behavior in different countries. Alongside questions about background information such as age and education and a variety of health-related and demographic questions, the Arabic version of the MeSHe survey includes the Arabic version of DUDIT, produced in cooperation with the original developers of this instrument at Karolinska Institute, Stockholm, Sweden. In Morocco, participants were recruited from three different settings with various pattern of drug use: (a) substance-dependent out-patients from a medical and psychological prevention center, (b) inmates with high possibility of their criminal behavior coexisting drug use problem, and (c) high school students with possible no drug use problem; each samples helping in the assessment of the discriminative validity of the DUDIT. Participation of the MeSHe study is always voluntary and involves the anonymous completion of the survey as self-reported questionnaire. In average (across all sub-samples), the completion of the whole MeSHe survey took about 45 min.

## Study Populations

### Clinical Sample

The sample of 61 substance dependent patients was recruited from the medical and psychological prevention center in Tangier, Morocco. Participation was 100% from this center which is operating on an outpatient basis. Based on clinicians’ assessments, all participants met the DSM-IV criteria for substance use disorder (SUD). It should be noted that no differential diagnoses were provided for research due to patient–doctor confidentiality. The number of female subjects (*n* = 7) in this clinical sample was too low to be able to perform any reliable statistical analysis, which would have been necessary based on the previously published gender sensitivity of DUDIT. Therefore, only male subjects (*n* = 54) were included in the present study. This sample had a mean age of 38.37 (SD = 8.29, min = 19 max = 56), the mean education dropout age was 15.55 (SD = 4.66), 72.2% achieved elementary or secondary school, 22.2% completed high school, 3.7% achieved higher education, and 1.9% were unable to achieve any qualification. A total of 59.3% of the patients were unemployed.

### Prison Sample

Data were collected from the male prison institution in Meknes, Morocco. Random recruitment, assured by the prison administration, was performed with exclusion of those who lacked the academic skills required to understand and answer the Arabic questionnaire. The initial sample size included 177 prisoners. Eight respondents (4.5%) were excluded due to missing information about their age, resulting a final sample of 169 inmates, which is approximately 7% of the prisoners who were incarcerated during the specific period the data collections took place. This sample had a mean age of 30.88 (SD = 10.66, min = 15 max = 92). The mean education dropout age was 17.75 years (SD = 3.76); 44.4% successfully achieved elementary or secondary school, 33.1% completed high school, 20.1% achieved higher education, 1.2% did not achieve any qualifications, and 1.2% were coded as missing. Employment status showed that 18.9% of respondents were unemployed.

### Students

Students from the “Sharif IDRISSI” high school in Tetouan, Morocco, were also asked to reply anonymously to our survey. Previously, the high school’s parents association approved the use of the survey based on anonymous and voluntary participation. At each grade (first, second, and third grades), there were four classes in the school, of which two were randomly selected to participate in the study. In each class, the study was thoroughly explained, and the voluntary and anonymous participation was emphasized. A member from the researcher team and co-author (BZ) was present in the classes while students completed the survey. No clinical backgrounds were available for this sample. Ninety-six students returned the questionnaire (representing 56.5% of the entire student population of these six classes). In the present analyses, only male subjects (*n* = 53) were included, with a mean age of 17.26 (SD = .68).

## Measures

The DUDIT is a screening instrument composed of 11 items identifying consumption patterns and different problems related to the use of drugs in general or clinical populations. The scoring of DUDIT is based on two approaches: items 1 to 9 are scored on a five-point Likert scale, while items 10 and 11 are scored on three-point scale. The DUDIT score is calculated by summing the scores on all items, engendering a maximum score of 44 points. Cutoffs for screening of drug-related problems (≥6 for man) and of drug dependence (≥25 points) were established in the original Swedish version of the questionnaire [17].

## Official Translation of DUDIT

The translations were performed in two steps: the first step was to translate DUDIT from English to Arabic, and the second step was a back-translation by an independent translator from Arabic to English. In 2014, after several adjustments, the developer (Berman and colleagues) approved a final version of the Arabic DUDIT, which is now downloadable at http://www.emcdda.europa.eu/attachements.cfm/att_236059_EN_DUDIT_Arabic_final.pdf.

## Ethical Considerations

The present study is in agreement with the Helsinki declaration. All participants received a written and oral presentation of the study and its aims. They were assured that their answers would not have any effect on their present sentence (in prison), treatment plan (in clinical population), or academic performance (students) and that no responses could be traced back to the individual level. All answers were recorded on an anonymous response sheet. Those who were not willing to participate could simply leave or not enter the questionnaire room, which provided a private, peaceful environment for answering the “Mental and Somatic Health without borders” (MeSHe) survey.

## Statistical Analysis

Sample characteristics were described via the use of descriptive statistics, including means and standard deviation. Principal factor analysis with oblique rotation was used to assess the internal structure of the instrument; the factorability of the data was assessed simultaneously by Bartlett’s test of sphericity and the Kaiser–Meyer–Olkin (KMO) measure of sampling adequacy. Internal reliability was tested using Cronbach’s alpha coefficient; we also included inter-item, total item, and item-rest correlations (IRC). We have followed George and Mallery (2003) rules of thumb for interpretation of the alpha values:“ ≥ .9 = Excellent, ≥.8 = Good, ≥.7 = Acceptable, ≥.6 = Questionable, ≥.5 = Poor, and ≤.5 = Unacceptable” (p. 231) [31]. For the interpretation items’ factor loading, the following rules very applied: “ ≥.7 = Excellent, ≥.6 = Very good, ≥.5 = Good, ≥.4 = Fair, ≥.3 = Poor” (p. 649) [32]. External validation was performed by the Mann–Whitney *U* test to analyze the difference between the clinical sample and young adults. Effect size (*r*) was calculated between young adult and clinical samples by dividing the *Z* values by the square root of *n* (number of cases), while Cohen’s criteria [33] for effect sizes were applied. Logistic regression was undertaken using group membership (dichotomous variable: clinical sample and young adults) as the dependent variable and the DUDIT score as the predictor variable in a model. We then used the receiver operating characteristic (ROC) analysis to evaluate sensitivity, specificity, and cutoff scores. The AUC and the ROC curve were defined to assess validity of the instrument by comparing the DUDIT scores with DSM-IV diagnosis of substance use disorder. A logistic regression model was used to determine the predictive capacity of the DUDIT scores (independent variable) to identify dichotomous group membership category (dependent variable where existing SUD diagnosis is coded as 1 and the non-existing SUD diagnosis is coded as 0). All statistical analyses were executed by SPSS for Windows version 21.0.

## Results

### Factorial Validity

The 11 DUDIT items were subject to principal factor analysis and an assessment for suitability of data was performed. The resulting Kaiser–Meyer–Olkin value was .93, while Bartlett’s test of sphericity reached statistical significance, supporting the factorability of data. Further, the scree plot revealed a break just after the first factor with an eigenvalue >1 (7.36), explaining 66.9% of the variance. The following eigenvalue was .75 and accounted for just 6.81% of the total variance. Factor loadings for all items ranged from .66 (item 10) to .87 (item 1) (see Table 1).

**Table 1:** Factor loadings for DUDIT items (*N* = 240)

DUDIT		Factor 1
1	How often do you use drugs other than alcohol?	.86
2	Do you use more than one type of drug on the same occasion?	.68
3	How many times do you take drugs on a typical day when you use drugs?	.82
4	How often are you influenced heavily by drugs?	.79
5	Over the past year, have you felt that your longing for drugs was so strong that you could not resist it?	.83
6	Has it happened, over the past year, that you have not been able to stop taking drugs once you started?	.82
7	How often over the past year have you taken drugs and then neglected to do something you should have done?	.78
8	How often over the past year have you needed to take a drug the morning after heavy drug use the day before?	.85
9	How often over the past year have you had guilt feelings or a bad conscience because you used drugs?	.86
10	Have you or anyone else been hurt (mentally or physically) because you used drugs?	.66
11	Has a relative or a friend, a doctor or a nurse, or anyone else, been worried about your drug use or said to you that you should stop using drugs?	.79

*DUDIT* Drug Use Disorders Identification Test

### Internal Consistency

Cronbach’s alpha was calculated for DUDIT in the total study population, showing excellent internal consistency (.95). The Cronbach’s alpha coefficient was also calculated within each group, proving a stable and strong correlation between the items in each sample (.94 in the inmates’ sample, .89 in the clinical sample and .94 in the young adults). The range of item-total correlations was between .70 and .88. Additionally, item-rest correlations were all above .65, which shows that items highly correlate with the scale. Table 2 displays the inter-item, total item, and item-rest correlations.

**Table 2:** Inter-item, total-item, and item-rest correlations (IRC) for DUDIT (*N* = 240)

DUDIT items		1	2	3	4	5	6	7	8	9	10	11	IRC
1	How often do you use drugs other than alcohol?	1											.84
2	Do you use more than one type of drug on the same occasion?	.65	1										.66
3	How many times do you take drugs on a typical day when you use drugs?	.80	.69	1									.80
4	How often are you influenced heavily by drugs?	.74	.57	.66	1								.77
5	Over the past year, have you felt that your longing for drugs was so strong that you could not resist it?	.68	.47	.63	.64	1							.81
6	Has it happened, over the past year, that you have not been able to stop taking drugs once you started?	.62	.52	.65	.66	.75	1						.79
7	How often over the past year have you taken drugs and then neglected to do something you should have done?	.61	.54	.63	.56	.72	.72	1					.75
8	How often over the past year have you needed to take a drug the morning after heavy drug use the day before?	.68	.58	.71	.63	.73	.81	.71	1				.83
9	How often over the past year have you had guilt feelings or a bad conscience because you used drugs?	.78	.54	.66	.69	.69	.67	.65	.70	1			.84
10	Have you or anyone else been hurt (mentally or physically) because you used drugs?	.59	.43	.44	.58	.57	.50	.44	.53	.64	1		.65
11	Has a relative or a friend, a doctor or a nurse, or anyone else, been worried about your drug use or said to you that you should stop using drugs?	.69	.46	.63	.58	.70	.60	.60	.67	.77	.59	1	.77
DUDIT score		.88	.72	.84	.82	.84	.83	.79	.86	.88	.70	.82	

All correlations are significant at *p* < .001

*DUDIT* Drug Use Disorders Identification Test

### Predictive Validity

**Fig. 1:**
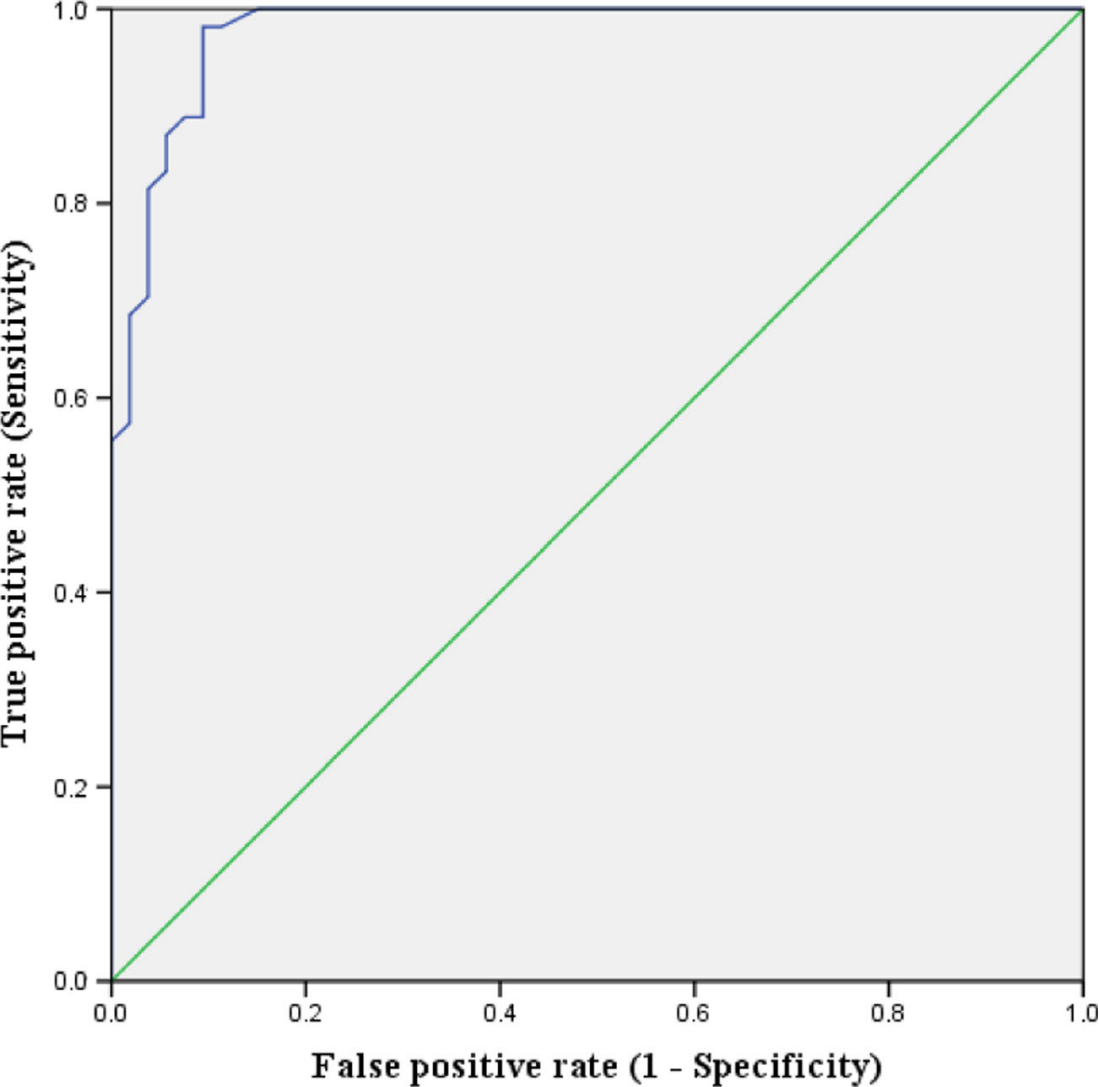
Receiver operating characteristic (ROC) curve for DUDIT score (independent variable) and group membership (dependent variable). Area under the curve (AUC) was .98 (*p* < .001, CI = .95–1)

The mean DUDIT score for the clinical sample diagnosed with SUD (*N* = 54) was 24.54 (SD = 12.05); this was significantly (*p* < .001) higher than in the young adult sample with no clinical SUD diagnosis (*N* = 53) (*M* = 1.34; SD = 4.43, *U* = 64.000). The difference had a large effect size (Cohen’s *r* = .85). A logistic regression model predicting group membership (SUD or no SUD) was statistically significant (χ2 (1, *N* = 107) = 93.52), indicating that the reported DUDIT score correlated highly with the existence of a SUD diagnosis. The overall model explained between 58.3% (Cox and Snell R square) and 77.7% (Nagelkerke R squared) of the variance of group membership and showed that the DUDIT score was effective in terms of correctly classifying the clinical sample with SUD and individuals without existing SUD in 88.8% of cases. Predictive validity was examined using ROC analysis, where AUC reached .98 (*p* < .001, CI = .95–1.00) (Fig. 1). Optimal sensitivity and specificity (.98 and .90, respectively) matched a cutoff score of 3 (Table 3).

**Table 3:** Specificity, sensitivity, and cutoff scores for the DUDIT (*N* = 107)

Cutoff scores	Sensitivity	Specificity
1	1	.42
2	.99	.87
3^a^	.98	.90
4	.94	.91
5	.89	.92
6^b^	.88	.93
7	.86	.94
8	.84	.94
9	.82	.95
25^c^	.28	1

^a^Optimal sensitivity and specificity in the Arabic DUDIT for identification of drug dependence

^b^Suggested cutoff for drug-related problems [17]

^c^Suggested cutoff for drug dependence [17]

## Discussion

This study has shown that the Arabic translation of DUDIT has a high validity and reliability to identify individuals with substance use/abuse in Arabic-language samples.

In matters of factorial validity, the one factor solution revealed by principal axis factoring supports the statement that the Arabic version of the DUDIT assesses a one-dimensional construct. The factor loading ranged between “very good” and “excellent.” All items loaded highly in the main factor, and those most strongly correlated with it concerned the frequency of substance use (items 1 and 3), developing dependence (item 5), uncontrolled use (item 6), and physical and psychological discomfort (items 8 and 9). A similar one-factor construct was previously identified in the Turkish, Dutch, and American validation studies [23, 26, 29]. The original Swedish study among a sample of drug users suggested a three-factor solution, whereas only two factors were reported in the general population [17]. Other validation studies also showed a two-factor structure [22, 28]. The present study is the fourth (after the Turkish, Dutch, and American studies) to find a one-dimensional construct of the instrument. The main similarities between these four studies were sample characteristics such as male gender predominance and relatively heterogeneous samples including SUD patients and inmates. Among these four studies, the country with highest GDP (gross domestic product) per capita was the USA, followed by the Netherlands, Turkey, and finally Morocco [34]; these countries also have strong cultural differences and differing attitudes to drug use and treatment of addiction.

Generally, the Arabic version of the DUDIT showed excellent reliability and high external validity, and psychometric properties were similar to those previously reported for other language versions [17, 21–23, 27, 29, 30]. Internal consistency calculated for the total study population, as well as for the separate samples, revealed excellent reliability (Cronbach’s alpha close to or above .90), in accordance with previous findings [22, 24, 26, 28–30]. Predictive validity based on ROC analysis also showed excellent results. The AUC of .98 reflects a high concurrent validity and indicates an ideal fit between the DUDIT score and the DSM-IV diagnosis; this indicates that the DUDIT can be considered “excellent” at separating drug users from non-drug-users. In order for a screening instrument to be considered clinically useful, sensitivity and specificity values must be above .80 [35]. The optimal sensitivity and specificity were coupled to a cutoff value of 3 (sensitivity .98 and specificity .90). This is lower than the value reported by Berman et al. [17] in a Swedish cohort sample (cutoff for defining drug addiction ≥25, with a sensitivity of .90 and a specificity of .88), but comparable to the value found in a Hungarian sample [22] (cutoff for defining drug addiction ≥2.1, with sensitivity of .95 and specificity of .81). It is important to point out that the original article [17] with the cutoff of 25 or more referred to DSM-IV/ICD-10 diagnoses based on a full diagnostic interview. The original Swedish study was conducted in samples of hospitalized or incarcerated subjects suffering from drug abuse or addiction, while Matuszka et al. [22] and other authors (including the present study) worked with a study population that included less severe substance use problems and referred to problematic drug use including hazardous and harmful use. In our study, each of the patients in the clinical sample had a SUD diagnosis based on DSM-IV criteria, but we did not have enough information to be able to differentiate subgroups according to the severity of drug abuse. The cutoff would obviously be much lower for hazardous and harmful use than for dependence or abuse. This important difference in the study populations is reflected in the mean scores in DUDIT. While students had very similar mean scores in both the Hungarian and the present Moroccan sample (1.39 and 1.34, respectively), we found clear differences in our clinical samples. The mean score of the clinical sample on DUDIT was the lowest in the Hungarian sample (*M* = 14.07 for mandatory drug treatment program participants and *M* = 17.71 for outpatient treatment program participants) [22], followed by that in the Moroccan SUD sample (*M* = 24.5) and the original Swedish report (32.7 points) [17]. One explanation of this is that the low cutoff score could be due to the differences in sample characteristics. The low cutoff could also reveal eventual cultural differences in attitudes to drug abuse and addicts, and emphasize the need for nationally validated drug inventories.

In our clinical sample, seven patients had between two and six points on the DUDIT, which should be impossible considering that each of these patients had a SUD diagnosis according DSM-IV and that they were hospitalized for their drug use problem. However, because the study was a validation of a self-report instrument, we did not exclude those persons from the analyses. Self-report as a method includes the possibility of misunderstanding the questions and the possibility of “under-scoring” based on shame or fear of admitting the truth about something (drug use, in our case). This is supported by the fact that when we use the suggested cutoff (≥3 points) to select those with drug use problems in our student sample (in average 17 years old), six students (11%) were identified. This rate is comparable to Swedish ninth-class boys (on average 15 years old), where 7% of whom reported use of narcotics [36].

## Limitations

This study has certain limitations. One is the exclusion of females in the study due to male gender predominance, which could have an effect on the results. Even though it can be useful and acceptable to only report results for males, further assessment focusing on female gender is required in order to acquire better insight regarding patterns of use among women. Furthermore, the absence of clinical diagnoses in both offender and student samples raises concerns about sensitivity and specificity of the instrument; future studies on predictive validity in a clinically diagnosed sample are highly recommended.

## Conclusion

The Arabic version of DUDIT has excellent reliability and high validity. These psychometric properties justify the use of this instrument for drug use assessment and for testing the treatment process in different settings, making it simple for clinicians and researchers to collect data from targeted groups. Moreover, our findings emphasize the need to investigate the cultural aspects of mental ill health and the use of locally adapted and validated measures in research.
